# Gabapentin reduces painful bladder hypersensitivity in rats with lipopolysaccharide‐induced chronic cystitis

**DOI:** 10.1002/prp2.697

**Published:** 2020-12-19

**Authors:** Masaru Yoshizumi, Chizuko Watanabe, Hirokazu Mizoguchi

**Affiliations:** ^1^ Department of Physiology and Anatomy Faculty of Pharmaceutical Sciences Tohoku Medical and Pharmaceutical University Sendai Japan

**Keywords:** bladder pain syndrome, chronic cystitis, gabapentin, interstitial cystitis, lipopolysaccharide

## Abstract

Although interstitial cystitis/bladder pain syndrome (IC/BPS) is a chronic condition causing bladder pain and urinary symptoms, effective treatments have not been established. The aim of this study was to adapt a chronic cystitis model in rats using lipopolysaccharide (LPS), which reflects IC/BPS pathology, and characterize the model's histological and behavioral effects. Furthermore, we investigated the effect of an α_2_δ subunit ligand, gabapentin (GBP), on bladder hypersensitivity of rats with chronic cystitis. Cystitis models were created by repeated intravesical injections of LPS. In the histological examination, the LPS‐injected group had greater inflammatory response, fibrosis, and abnormally thick re‐epithelialization. In the LPS‐injected group, LPS prompted hyperalgesia in both the lower abdomen and hind paw regions after day 1 of the first injection compared with the saline‐injected controls, without any recovery for 21 days at least. During cystometry, the LPS‐injected group showed bladder hyperactivity at all times. Systemic administration of GBP reduced cystitis‐related pain due to chronic inflammation and reduced the increased frequency of voiding in the LPS‐injected group. These results suggest that repeated intravesical injections of LPS induce long‐lasting bladder inflammation, pain, and overactivity in rats, while GBP is effective in the management of those symptoms in this chronic cystitis model. The current study identifies a relatively simple method to develop an animal model for chronic cystitis and provides evidence that GBP may be an effective treatment option for patients with IC/BPS.

## Graphical abstract

This study shows the repeated intravesical injections of lipopolysaccharide (LPS) could be used to generate a model of chronic cystitis in rats, which show signs of long‐lasting bladder inflammation, pain, and overactivity. Gabapentin, a neuromodulator, effectively inhibited bladder pain and overactivity in the LPS‐induced chronic cystitis rats.

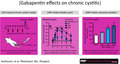

**Abbreviations**
ABHCA: 3‐exo‐aminobicyclo [2.2.1] heptane‐2‐exo‐carboxylic acid
Bar: Barrington's nucleus
BOO: bladder outlet obstruction
CPA: cyclophosphamide
CRH: corticotropin‐releasing hormone
GBP: gabapentin
H&E: hematoxylin and eosin
IC/BPS: interstitial cystitis/bladder pain syndrome
ICI: intercontraction interval
IL‐1β: interleukin‐1β
LC: locus coeruleus
LPS: lipopolysaccharide
MVP: maximum voiding pressure

## 1 INTRODUCTION

Interstitial cystitis/bladder pain syndrome (IC/BPS) is a chronic bladder inflammation characterized by bladder and pelvic pain, and urinary symptoms, such as urinary frequency, urgency, and nocturia.^1^, ^2^ IC/BPS primarily affects women, but it can also occur in men over a broad age range, and severely affect the patient's quality of life.^3^, ^4^ The etiology of IC/BPS is still not completely understood, and effective drug treatments have not been established.^2^ Although IC/BPS models have been evaluated by a variety of methods,^5^, ^6^, ^7^, ^8^, ^9^, ^10^, ^11^ many of these are acute inflammation models. The durations of cystitis‐related pain and bladder overactivity in these models are short, which are not consistent with the pathophysiology of chronic conditions displayed in patients with IC/BPS. Therefore, it is necessary to confirm the efficacy and safety of new therapies using an appropriate animal model with characteristics similar to those of the condition in human beings.

Lipopolysaccharide (LPS) is the main outer‐membrane component of the gram‐negative bacteria, including *Escherichia coli*, and acts as a common virulence factor.^12^ Intravesical instillation of LPS induces an inflammatory response mediated by the activation of mast cells, production of cytokines, and recruitment of leukocytes to the mucosal surface of the bladder, similar to that observed in patients with IC/BPS.^13^, ^14^, ^15^ Although repeated intravesical injections of LPS have reportedly increased the expression of macrophage migration inhibitory factor (a pro‐inflammatory cytokine) in both the bladder and the lumbosacral spinal cord,^14^ symptoms of bladder pain and bladder overactivity have not been demonstrated.

Gabapentin (GBP), an antiepileptic drug, has been effectively used in various chronic pain treatments and is especially suitable for neuropathic pain.^16^, ^17^, ^18^, ^19^, ^20^ We and others have previously demonstrated that GBP acts as a neuromodulator by selectively binding to the α_2_δ subunits of voltage‐gated Ca^2+^ channels in various regions throughout the central nervous system, such as the locus coeruleus (LC) within the brainstem, and the spinal dorsal horn in rats after peripheral nerve injury.^17^, ^21^, ^22^, ^23^ As a result, it demonstrates a therapeutic effect on chronic pain by inhibiting the primary afferent traffic and excitation of the spinal nociceptive neurons.^24^ Additionally, GBP has been observed to have a beneficial effect on bladder pain and overactivity related to IC/BPS.^25^, ^26^ In rodent acute cystitis models, a recent study reported that GBP could reduce detrusor overactivity and the visceral nociception,^27^ but another study had failed to identify any effect.^28^ Therefore, it is necessary to evaluate the effectiveness of GBP in chronic cystitis models with IC/BPS symptoms.

The current study confirmed whether repeated intravesical injections of LPS cause sustained bladder pain‐related behavior and bladder overactivity in rats. Thereafter, we tested the effects of GBP on those symptoms in a rat model of LPS‐induced chronic cystitis.

## 2 MATERIALS AND METHODS

### 2.1 Animals

Adult female Sprague‐Dawley rats (Japan SLC, Hamamatsu, Japan), weighing 200‐300 g, were used in this study. The animals were housed in a room maintained at 22‐24℃ and 50%‐60% relative humidity with an alternating 12‐h light–dark cycle. Food and water were available ad libitum. All animal procedures were approved by the Committee of Animal Experiments, Tohoku Medical and Pharmaceutical University and were performed in accordance with the NIH Guide for the Care and Use of Laboratory Animals.

### 2.2 Induction of LPS‐induced cystitis

Rats were anesthetized with 2% isoflurane (Pfizer Inc). A PE‐50 polyethylene tube (Becton Dickinson) was inserted into the bladder via the urethra to empty the bladder, and 0.5 mL of LPS (*E*.* coli* O55:B5, Sigma‐Aldrich) at 1 mg/mL in sterilized saline was infused intravesically and remained in the bladder for 30 min. After the LPS exposure period, the bladder was rinsed once with saline and allowed to drain freely from the open catheter end. Chronic cystitis was induced by intravesical LPS performed every 24 hours for 4 days in the same manner. The control group rats received 0.5 mL of saline into the bladder.

### 2.3 Histology

The bladders were fixed in 4% paraformaldehyde at 7, 14, and 21 days after the first LPS injection. The bladders of the control rats were collected 7 days after the first saline injection. Tissues were frozen and cut on a cryostat at 5‐μm thickness, and then stained with hematoxylin and eosin (H&E) and Masson's trichrome staining for the histology of inflammatory cell infiltration and fibrosis, respectively. Gross histologic observations were performed using a microscope system (BZ‐X800; Keyence).

### 2.4 Drugs and administration

For oral administration, GBP (Tokyo Chemical Industry) was dissolved in distilled water and administered using a feeding tube (30‐300 mg/6 mL per kg). For intravenous administration, GBP was dissolved in sterilized saline and injected (10‐300 mg/3 mL per kg) at the rate of 1.2 mL/h.

### 2.5 Behavioral studies

Hypersensitivity response to the lower abdomen and hind paw was assessed using calibrated von Frey filaments (Danmic Global, LLC). Rats were placed individually in a small acrylic cage with a wire mesh floor and acclimated to the experimental environment for 1 hour. In the abdominal stimulation, eight von Frey filaments exerting from 2 g to 60 g were used to assess the pain threshold. Tactile sensitivity of the region between the anus and urethral opening was assessed by applying the filaments perpendicularly to the surface of the skin. To prevent the wind‐up effects of desensitization, repeated stimulation of the same location was avoided. Behaviors considered as positive response to filament stimulation were sharp retraction of the abdomen, immediate licking or scratching of the area of filament stimulation, and jumping. For the hind paw stimulation, eight von Frey filaments exerting 0.6‐26 g were used to assess the pain threshold. Filaments were applied to the plantar surface of the hind paw, and a brisk paw withdrawal was considered as the positive response. Withdrawal threshold was determined using an up‐down statistical method.^29^

### 2.6 Cystometric studies

Cystometry was performed as we had reported previously.^30^ Rats were anesthetized using 2% isoflurane, and a midline abdominal incision was made to expose the bladder. A PE‐50 polyethylene tube with a fire‐flared tip was implanted into the bladder dome for bladder filling and pressure recording 2 days before the experiments. A PE‐10 polyethylene tube (Natsume Seisakusho) was inserted into the right jugular vein for intravenous drug administration. After surgery, rats were placed in a Ballman restraining cage (Natsume Seisakusho) and were allowed to recover from anesthesia for 1 hour. Physiological saline was infused at room temperature (22‐24℃) into the bladder at a rate of 2.4 mL/h. Intravesical pressure was recorded using a force transducer, quad bridge amplifier FE224 (ADInstruments), and PowerLab data‐acquisition system with LabChart Pro (ADInstruments). During the course of saline infusion, before drug administration, three voiding cycles were recorded as the control values, and each parameter was averaged.

### 2.7 Statistics

Data were presented as the means ± SEM. Differences in the withdrawal threshold between the groups were analyzed using one‐ or two‐way analysis of variance followed by either the Dunnett's test or Bonferroni test. The commercial software GraphPad Prism version 7 (GraphPad Software) was used to calculate statistical significance. *P* < .05 was considered significant. A parametric test (unpaired *t*‐test) was used to test for differences in the cystometric variables between the two groups.

### 2.8 Nomenclature of targets and ligands

Key protein targets and ligands in this article are hyperlinked to corresponding entries in http://www.guidetopharmacology.org, the common portal for data from the IUPHAR/BPS Guide to PHARMACOLOGY (Harding et al, 2018),^31^ and are permanently archived in the Concise Guide to PHARMACOLOGY 2019/20: Ion channels (Alexander et al, 2019).^32^

## 3 RESULTS

### 3.1 Bladder histology

**FIGURE 1:**
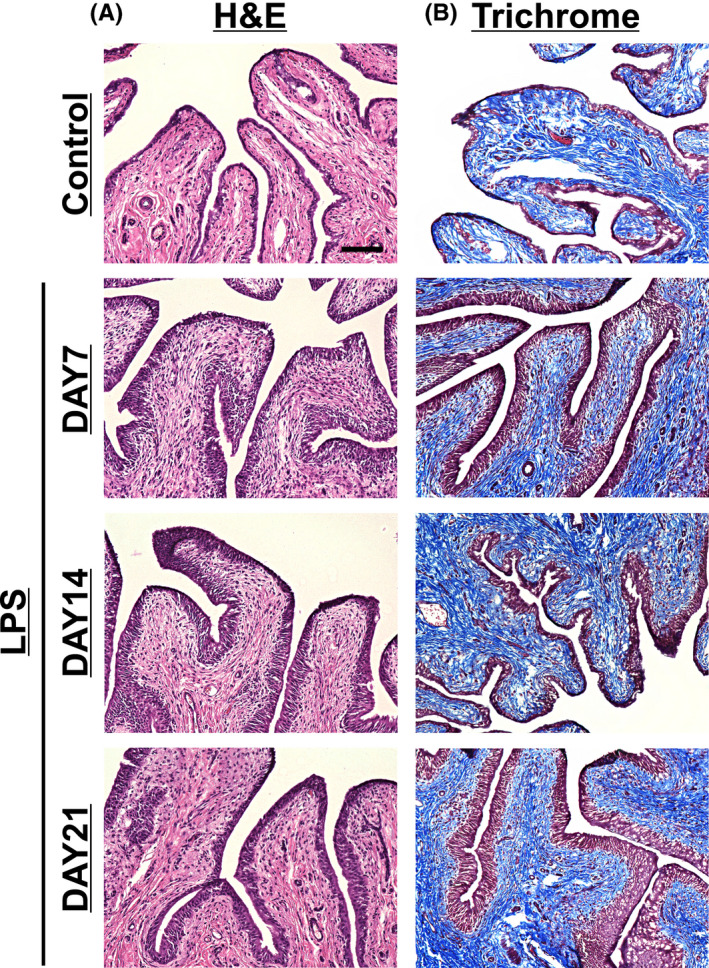
Histological findings of LPS‐induced chronic bladder inflammation. The bladders of rats were instilled with saline or LPS in saline (1 mg/mL) at every 24 hour for 4 days. At 7, 14, and 21 days after the first LPS injection, the bladders were collected and processed for histological hematoxylin and eosin (H&E) and Masson's trichrome staining. The bladders from the control rats were collected at 7 days after the first saline injection. A, In the H&E staining, LPS‐induced cystitis showed inflammatory cell infiltration and urothelial hyperplasia. B, LPS‐induced cystitis showed accumulation of fibrous tissue in the submucosal layer by Masson's trichrome staining. Magnification ×20, scale bar = 100 μm

Chronic inflammation induced from repeated intravesical administrations of LPS was observed even 21 days after the first injection (Figure 1). In the H&E histological examination, LPS‐induced cystitis presented a mixed inflammatory cell infiltrate, predominantly comprised of macrophages and lymphocytes, increased numbers of urothelial cells, and abnormally thick re‐epithelialization compared with saline‐injected rats (control group) (Figure 1A). Masson's trichrome staining in the LPS‐injected group showed an increase in bladder tissue fibrosis, indicated by the blue coloring, compared with the control group (Figure 1B).

### 3.2 LPS‐induced bladder pain‐related behavior

**FIGURE 2:**
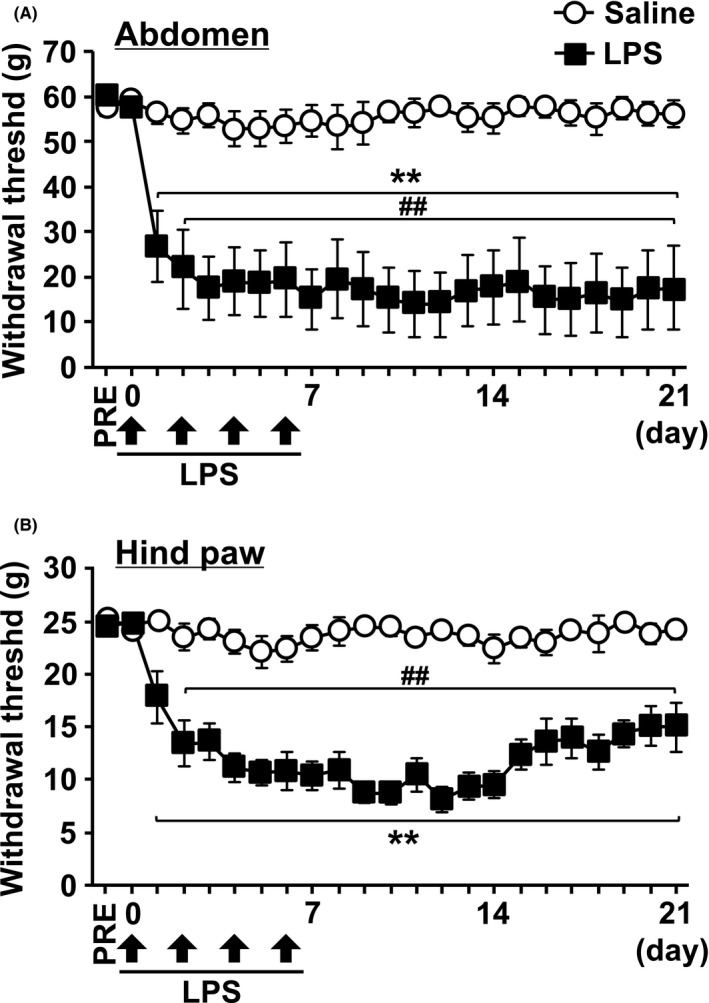
The time course of the mechanical threshold in chronic cystitis induced by repeated LPS in rats. The bladders of rats were instilled with saline or LPS in saline (1 mg/mL) at every 24 hours for 4 days. The von Frey filaments were applied to (A) the abdominal area close to the urinary bladder and (B) the plantar surface of the hind paw. The mechanical withdrawal threshold is presented over time. Each point represents the means ± SEM for eight rats. **P < .01 vs saline. ^##^ *P* < 0.01 vs pre values

At first, chronic cystitis due to repeated intravesical exposures to LPS was observed to induce persistent pain in rats (Figure 2). Compared with saline‐injected rats, the withdrawal thresholds in the abdomen (Figure 2A) and the hind paw (Figure 2B) were significantly reduced beginning at day 1 following the first injection of LPS and persisted until day 21 at least. The difference in body weight between the two groups was not significant (data not shown).

### 3.3 LPS‐induced increased bladder excitability

**FIGURE 3:**
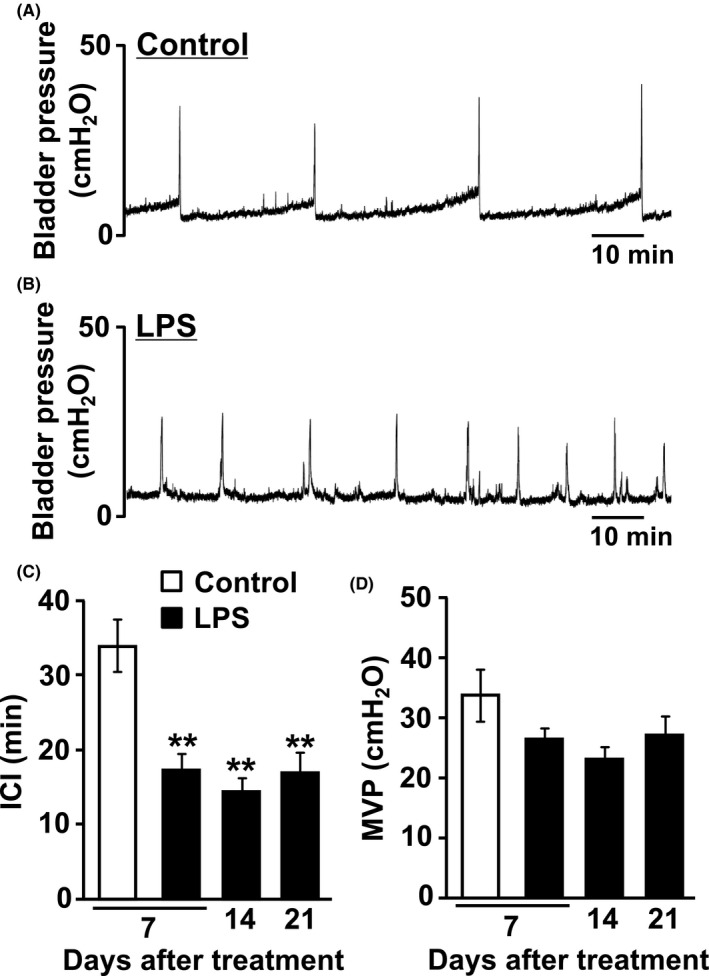
Representative continuous cystometrogram tracings from (A) control and (B) LPS‐injected group in conscious rats. Changes in the (C) intercontraction interval (ICI) and (D) the maximal voiding pressure (MVP) in control and LPS‐injected groups. Each point represents the means ± SEM in 6‐7 rats. **P < .01 vs control

Representative traces of the cystometrograms obtained after repeated intravesical instillation of saline and LPS are shown in Figure 3A and B, respectively. In the control group, during saline infusion, the intercontraction interval (ICI) and maximum voiding pressure (MVP) (n = 7) were 33.9 ± 3.5 minutes and 33.8 ± 4.3 cmH_2_O, respectively (Figure 3C and D). The LPS‐injected group had shorter ICI by 49.3% at 7 days (*P* = .0004, n = 7), 57.3% at 14 days (*P* = .0001, n = 6), and 49.9% at 21 days (*P* = .0005, n = 6) after the first LPS injection compared with the control group (Figure 3C). The MVP of the LPS‐injected group was slightly lower than that of the control group, although this was not significantly different (Figure 3D).

### 3.4 Effect of GBP on bladder pain‐related behavior in LPS‐induced cystitis

**FIGURE 4:**
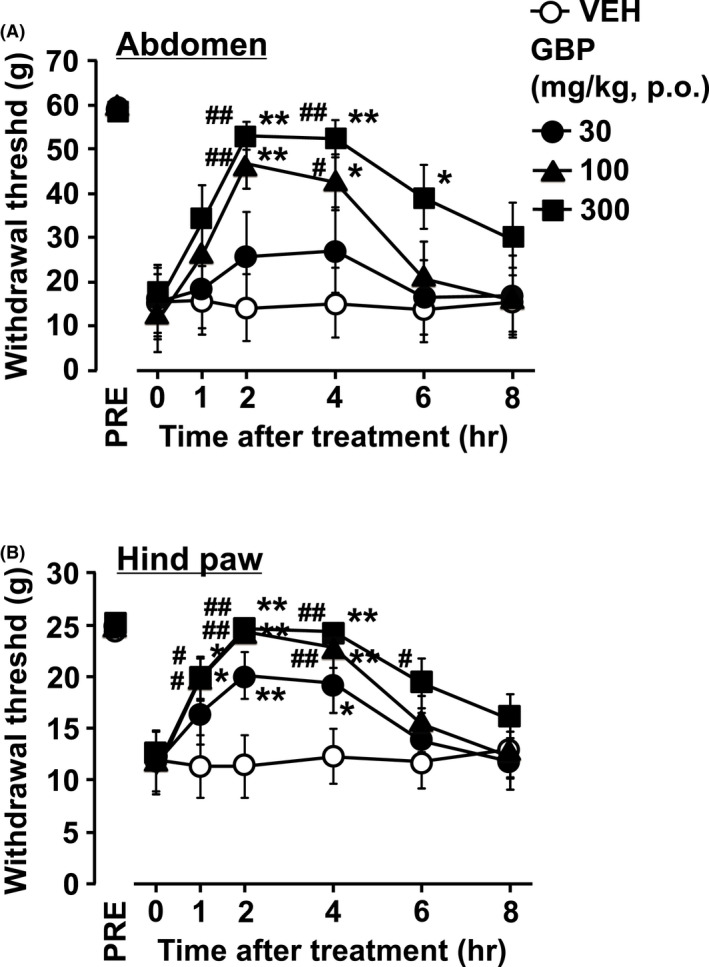
Effect of orally administered GBP on chronic cystitis‐related pain caused by LPS in rats. The von Frey filaments were applied to (A) the lower abdominal area close to the urinary bladder and (B) the plantar surface of the hind paw. At 7 days after the first intravesical injection of LPS (time 0), the rats were administered with GBP [30‐300 mg/kg, oral (p.o.)]. Values were obtained before the first LPS injection. The mechanical withdrawal threshold is presented over time. Each point represents the means ± SEM for eight rats. *P < .05, **P < .01 vs vehicle (VEH). ^#^ *P* < .05, ^##^ *P* < .01 vs time 0

The analgesic effect of GBP on cystitis‐related mechanical hyperalgesic behavior was tested at 7 days after the first LPS injection. Orally administered GBP (30‐300 mg/kg) suppressed mechanical hyperalgesia in the abdomen and the hind paw, evoked by LPS, in a dose‐dependent manner (Figure 4). In the abdomen (Figure 4A), oral GBP showed significant analgesic effects in doses of 100 and 300 mg/kg compared with that of the vehicle. In the hind paw (Figure 4B), oral GBP showed significant analgesic effects from 30 to 300 mg/kg compared with that of the vehicle. The peak effect of oral GBP was observed 2‐4 h after administration.

### 3.5 Effect of GBP on bladder overactivity in LPS‐induced cystitis

**FIGURE 5:**
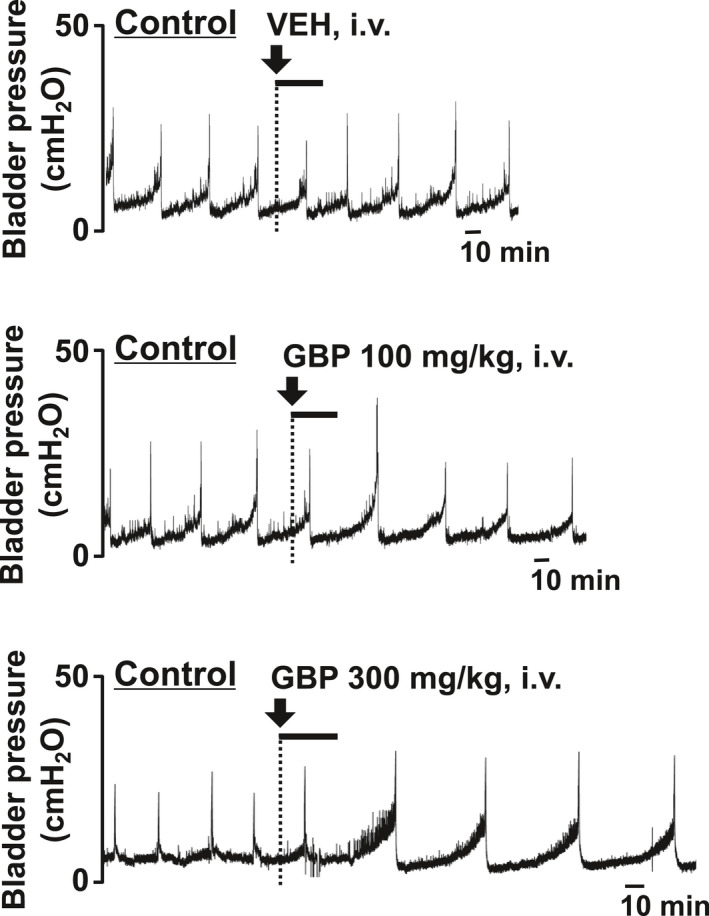
Representative continuous cystometrogram traces showing the effect of intravenous administration of GBP on the control group in conscious rats. Arrow indicates the timing of drug administration. VEH, vehicle

**FIGURE 6:**
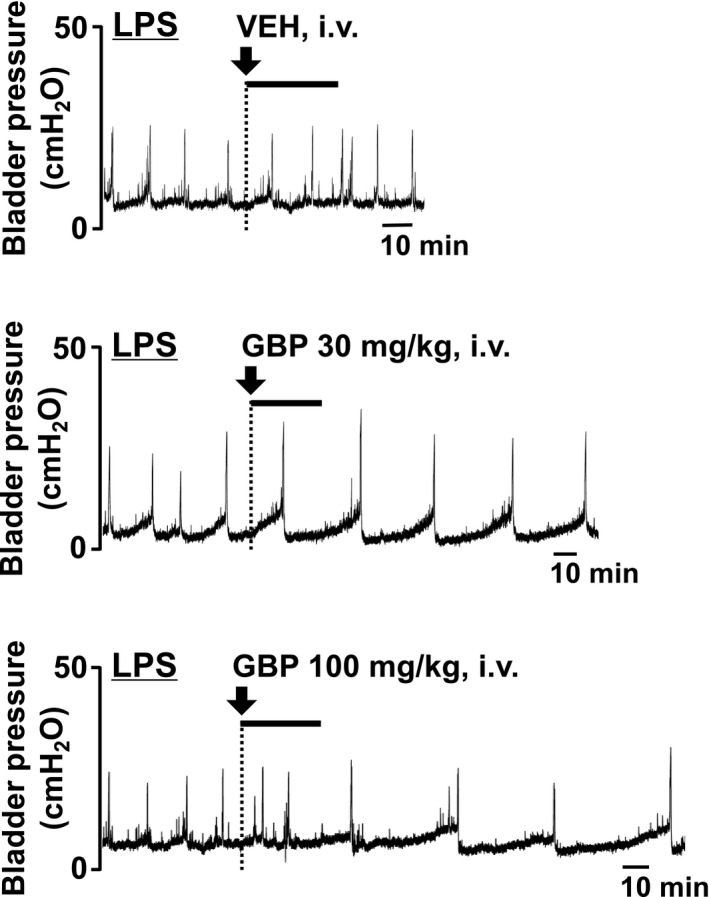
Representative continuous cystometrogram traces showing the effect of intravenous administration of GBP on LPS‐injected group in conscious rats. Arrow indicates the timing of drug administration. VEH, vehicle

**FIGURE 7:**
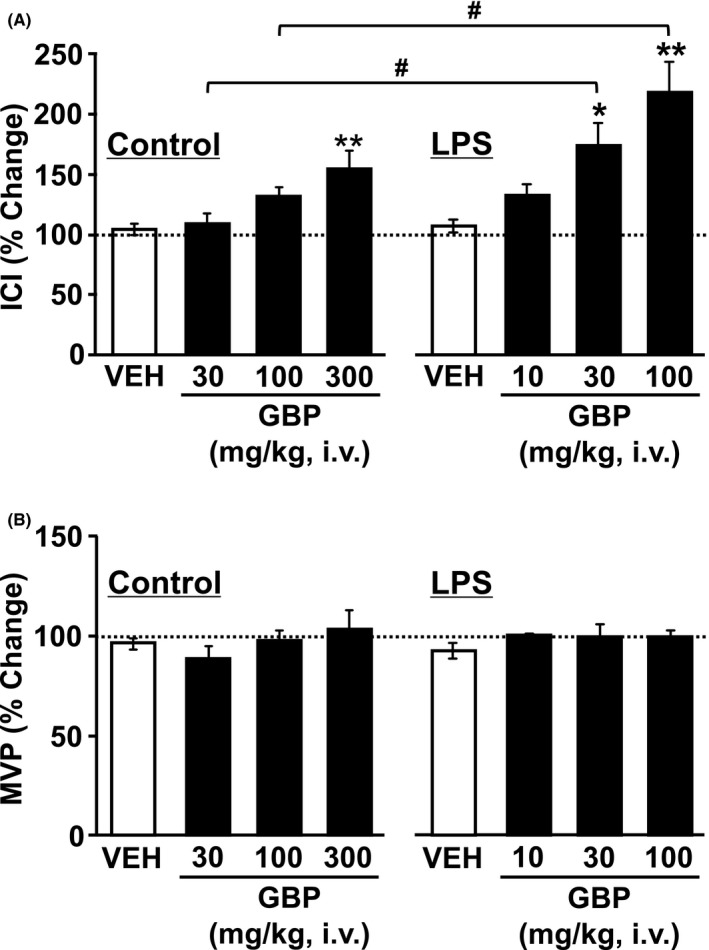
Effect of intravenously administered GBP on the (A) intercontraction interval (ICI) and (B) the maximal voiding pressure (MVP) in the control and LPS‐injected groups in conscious rats. At 7 days after the first intravesical injection of saline or LPS, rats were administered with GBP [10‐300 mg/kg, intravenous (iv)]. Responses are represented as % change, where the baseline response before administration is defined as 100%. Each point represents the means ± SEM in 5‐6 animals. *P < .05, **P < .01 vs vehicle (VEH). ^#^ *P* < .05 vs the values between the two groups at intravenous administration of 30 and 100 mg/kg GBP (unpaired *t*‐test)

To test the therapeutic effect of GBP on overactive bladder symptoms, the effect of GBP on bladder overactivity was examined 7 days after the first LPS injection. In both the control and LPS‐injected groups, intravenous administration of GBP (10‐300 mg/kg) caused a dose‐dependent prolongation of ICI (Figures 5, 6, 7) without changing the MVP (Figures 5, 6, 7). In the control group, intravenously administered GBP significantly prolonged ICI at a dose of 300 mg/kg compared with that of the vehicle (*P* = .0045, n = 5, Figure 7A). In the LPS‐injected group, intravenously administered GBP significantly prolonged ICI in doses of 30 and 100 mg/kg compared with that of the vehicle (*P* = .0271, n = 6 and *P* = .0005, n = 6, respectively; Figure 7A). The prolongation of ICI due to GBP (30 and 100 mg/kg) in the LPS‐injected group was significantly higher than that of the control group (*P* = .0156 and *P* = .0161, respectively; Figure 7A). In both groups, intravenous administration of GBP did not affect the MVP (Figure 7B).

## 4 DISCUSSION

The clinical symptoms related to IC/BPS include urinary frequency, urgency, nocturia, and pain in the lower abdomen and pelvic regions; however, its response to current therapies and drugs is poor.^2^ IC/BPS is one of the refractory syndromes and its evaluation in animal models that can imitate the clinical aspects of the syndrome in humans is essential for therapeutic development. Therefore, we created a chronic cystitis model in rats, which demonstrated a long‐lasting bladder inflammation, pain, and overactivity via repeated intravesical instillation of LPS. Additionally, the current study demonstrated that GBP, a drug used to treat chronic neuropathic pain, reduced bladder pain‐related behavior and overactivity in this chronic cystitis rat model.

Cyclophosphamide (CPA) is most commonly used in rodents for developing an experimental acute and/or chronic model for IC/BPS.^6^, ^8^, ^9^, ^33^, ^34^ However, CPA‐induced chronic models lead to deleterious effects, such as strong body weight loss associated with high mortality.^35^, ^36^ Adjusting CPA doses can reduce weight loss and mortality but CPA‐induced visceral pain does not appear sustained over long‐term CPA induction.^36^ LPS is an endotoxin that elicits inflammatory responses, and is used in various animal models of inflammation, including lower urinary tract infections. Similar to our study, previous studies in rodents have shown that intravesical LPS‐induced bladder inflammation, characterized by edema and leukocytic infiltration,^13^, ^14^ caused bladder pain and micturition dysfunction, which are typical symptoms seen in patients with IC/BPS.^15^, ^37^, ^38^ In fact, clinical reports have considered a high proportion of women having urinary tract infections at IC/BPS onset, and urinary tract infections resulting in the initiation of IC/BPS in some patients.^39^ The current study revealed chronic inflammation and bladder remodeling, including urothelial hyperplasia and prominent fibrosis, due to increased frequency of intravesical LPS instillation, consistent with studies in a chronic cystitis model induced by double instillation of protamine sulfate and LPS for 5 weeks.^40^ Although chronic cystitis models developed by injections of protamine sulfate and LPS once weekly for 5 weeks require long‐term manipulation, based on our observations, the LPS‐induced chronic cystitis rat model developed by a relatively simple method also demonstrated sustained pain and bladder overactivity. Furthermore, in our protocol, no severe body weight loss occurred.

Intravesical instillation of LPS induces the accumulation of inflammatory factors in the bladder, such as tumor necrosis factor‐α, interleukin‐1β (IL‐1β), and nerve growth factor, that cause sensitization of the afferent nerves.^10^, ^41^ Other studies have reported that inflammatory factors, including IL‐1β and several growth factors, result in bladder remodeling in the bladder outlet obstruction (BOO) model.^42^, ^43^ Therefore, the current findings suggest that continuous inflammation of the bladder with LPS could increase the hypersensitivity of the afferent nerves and induce bladder remodeling, leading to long‐lasting bladder pain and overactivity.

GBP has been used as an effective analgesic for neuropathic pain, and its mechanisms of analgesic effect have been extensively studied. The current study demonstrated that GBP not only reduced bladder pain, one of the refractory pains, but also markedly reduced bladder overactivity in LPS‐induced chronic cystitis rats. Accordingly, GBP has been shown to be useful in overactive bladder as well as in bladder pain. GBP has a high affinity for the α_2_δ subunit of the voltage‐gated Ca^2+^ channels (particularly the N‐type and L‐type),^21^, ^44^ and specific binding to this subunit is crucial for analgesic effects.^17^, ^45^ As spinal plasticity and sensitization, including upregulated α_2_δ subunits, play pivotal roles in pain amplification after peripheral nerve injury and inflammation,^17^ showing temporal correlation between α_2_δ subunit upregulation in dorsal root ganglia and allodynia,^46^, ^47^ most studies have focused on the peripheral afferents and spinal cord. In fact, animal models of bladder inflammation and BOO induce the upregulation of L‐, N‐, and T‐type Ca^2+^ channels in the bladder and spinal dorsal horn, which can result in hypersensitivity of the bladder.^48^ In the current study, GBP inhibited bladder overactivity in LPS‐induced cystitis rats, but its inhibitory effect was poor in the control rats. This is consistent with previous reports that suggested a lack of effect in the absence of hypersensitivity.^16^, ^18^ This has been emphasized by a study that demonstrated GBP to specifically inhibit Ca^2+^ currents in transgenic mice that overexpressed the α_2_δ‐1 subunits, but did not affect the wild‐type mice.^19^

Furthermore, GBP may produce pain attenuating effects by acting on the supraspinal regions to stimulate bulbospinal descending inhibition and alleviate neuropathic pain.^18^, ^20^ GBP, both systemically administered in vivo and locally applied to isolated brainstem slices in vitro, activated noradrenergic neurons in the LC.^49^ We and others have previously demonstrated that GBP reduces GABAergic activity in the LC by an interaction with the α_2_δ subunits, thereby increasing the activation of the descending pain inhibitory pathway to the spinal cord.^22^, ^23^ Interestingly, however, some α_2_δ subunit ligands, including 3‐exo‐aminobicyclo [2.2.1] heptane‐2‐exo‐carboxylic acid (ABHCA), fail to produce behavioral analgesia, indicating the involvement of additional mechanisms.^19^ We have previously demonstrated that GBP activates glutamate transporters and thereby facilitates glutamate‐induced glutamate release in cultured astrocytes, while ABHCA do not.^50^ An in vivo study also showed that GBP increased extracellular glutamate in the LC by astroglial glutamate transporter‐mediated mechanisms to stimulate descending inhibition aside from α_2_δ subunits in neurons.^51^ Thus, the antinociceptive effect of GBP is not solely responsible for the α_2_δ subunit, and the mechanism of action has not yet been definitively established.

In the micturition reflex, Barrington's nucleus (Bar; also known as the pontine micturition center), located rostral and ventromedial to the LC, has been identified as the major brain center regulating urination.^52^, ^53^, ^54^ The majority of neurons in Bar express a reporter for corticotropin‐releasing hormone (CRH)^55^, ^56^, ^57^, ^58^ co‐innervate spinal preganglionic neurons that control the bladder,^59^, ^60^ and LC neurons that provide noradrenaline innervation throughout the brain.^60^, ^61^ Furthermore, at least five separate population of neurons, including GABAergic and LC neurons, are located around Bar neurons.^57^ Since increased CRH expression in and around Bar has been reported to prolong the ICI in rodents,^62^, ^63^, ^64^ increased CRH may have an inhibitory effect on the micturition reflex.^65^ Furthermore, noradrenaline derived from the LC acts on the α_1_‐adrenergic receptors in the lumbosacral cord, and appears to contribute to the excitatory and inhibitory responses of the micturition reflex via the glutamatergic and glycinergic/GABAergic neurons in the spinal region.^66^ However, the exact central mechanisms by which GBP inhibits the micturition reflex in animal models of cystitis are still unknown. Further studies are ongoing at our laboratory to dissect the mechanisms of GBP to ameliorate both bladder pain and overactivity.

In summary, repeated intravesical injection of LPS induces consistent, reproducible inflammatory response and bladder remodeling including urothelial hyperplasia and prominent fibrosis in rats. This model maintained long‐lasting painful bladder hypersensitivity. The current study demonstrated that GBP inhibits not only bladder pain, but also bladder overactivity in LPS‐induced chronic cystitis rats. The LPS‐induced chronic cystitis model may be a simple and useful tool in the pathological and pharmacological study for chronic cystitis, such as IC/BPS, and given the clinical availability and established safety profiles, gabapentinoids, including GBP, are considered available as therapeutic drugs for the treatment of IC/BPS.

## DISCLOSURE

The authors have no conflict of interest to declare.

## AUTHORS CONTRIBUTIONS

Participated in research design: Yoshizumi and Mizoguchi.

Conducted experiments: Yoshizumi.

Performed data analysis: Yoshizumi.

Wrote or contributed to the writing of the manuscript: Yoshizumi, Watanabe, and Mizoguchi.

## Data Availability

Additional information and requests for data should be directed to the corresponding author, Masaru Yoshizumi. Please contact yoshizumi@tohoku-mpu.ac.jp.
